# The Association Between Oxidative Stress and the Progression of Heart Failure: A Systematic Review

**DOI:** 10.7759/cureus.55313

**Published:** 2024-03-01

**Authors:** Harsh V Chawla, Nikita Singh, Sangeeta B Singh

**Affiliations:** 1 Acute Medicine, Royal Cornwall Hospitals NHS Trust, Truro, GBR; 2 Internal Medicine, Albert Einstein College of Medicine, Jacobi Medical Center, New York, USA; 3 Biochemistry, Shaheed Hasan Khan Mewati Government Medical College, Nuh, IND

**Keywords:** hfref, hfpef, oxidative stress marker, a systematic review, antioxidant marker, chronic heart failure, oxidative stress, reactive oxygen species

## Abstract

Chronic heart failure (CHF) is a progressive multifactorial condition where the role of oxidative stress may have implications in the pathogenesis of the disease. Despite growing interest among researchers and clinicians, the limited, unorganized, and divergent findings regarding the association between oxidative stress and the progression of heart failure (HF) have prompted us to conduct this study. Drawing upon the evolving nature of this research domain, this study is one of the first of its kind to present a systematic and comprehensive overview of the existing evidence regarding the role of oxidative stress production in the progression of HF.

This study systematically reviews peer-reviewed empirical studies published in English, particularly focusing on the association between oxidative stress and the progression of HF. Parameters, such as publication year, study design, population demographics (size, age, and gender), types of HF, and characterization of markers in the existing studies, were reviewed. Following the Preferred Reporting Items for Systematic Reviews and Meta-Analyses (PRISMA) procedure, a thorough search was conducted on PubMed, Cochrane, Embase, and Sage databases, without any restrictions on the publication dates of articles, which yielded a total of 1,808 records on the association of oxidative stress production with clinical outcomes in HF patients. The analysis of the content of 17 articles offered a robust observation of this phenomenon, providing insights into the levels of oxidative stress, antioxidant markers, and the enzymes involved in the production of reactive oxygen species (ROS), and their association with the progression and severity of HF. The findings highlighted various knowledge gaps and future research priorities are recommended in the areas of interest and unexplored areas.

## Introduction and background

Chronic heart failure (CHF) or heart failure (HF), a cardiac disorder accompanied by structural and physiological abnormalities, is a growing concern and regarded as an epidemic in developed countries, with an increasing prevalence of around 1-2% among adults, which contributes to roughly 10% of mortality annually [1]. HF is signified by the neuroendocrine activation via the renin-angiotensin-aldosterone system (RAAS) as well as the sympathetic nervous system [2]. It is classified into three types: HF with reduced ejection fraction (HFrEF), HF with midrange ejection fraction (HFmrEF), and HF with preserved ejection fraction (HFpEF) [3]. HFrEF involves eccentric remodeling with dilation of the left ventricle (LV) following factors such as myocardial infarction (MI) and volume overload, ultimately leading to forward failure. On the other hand, HFpEF is characterized by concentric cardiomyocyte hypertrophy leading to impairment of ventricular relaxation or an increase in ventricular stiffness and an increase in LV diastolic filling pressures, often leading to backward congestion [4]. HFpEF patients account for approximately half of all patients diagnosed with HF [5]. Despite the improved outcomes based on effective treatment, the prognosis of HF is still poor with a five-year mortality rate of 25-50% [4]. Recently, the conceptual clarity about the pathophysiology and treatment of HFrEF and HFpEF has received increasing attention [6]. The pathophysiology of HF involves various mechanisms such as enhanced production of reactive oxygen species (ROS) [7].

Oxidative stress is caused by the imbalance of ROS generation, which includes superoxide anion, hydroxyl radicals, and oxidants [hydrogen peroxide (H_2_O_2_) and hypochlorous acid (HOCl)]. The detoxification of ROS by endogenous antioxidant enzymatic defense system (peroxiredoxin, superoxide dismutase, glutathione peroxidase, and catalase) is necessary for maintaining reduction-oxidation balance (redox balance) [8,9]. Oxidative stress is considered to be associated with the progression and development of various cardiac diseases, including HF [10-14]. The intensity of oxidative stress is assessed by its products (ROS) measured in plasma [15] and the ROS correlates with hypertrophy and LV dysfunction in HF [16]. ROS impairs calcium (Ca^2+^) homeostasis in the myocardium, resulting in cardiac arrhythmia and ventricular remodeling. This occurs through the activation of signaling pathways involved in the regulation of hypertrophy, metabolic defects, fibrosis, and programmed cell death [17].

Such roles of ROS are evident in studies that establish the association between the upregulation of several biomarkers with an impact on these factors that create stress and produce defects in subcellular organelles, like sarcoplasmic reticulum, myofibrils, sarcolemma, and mitochondria [18,19]. In the context of functional conditions, low concentrations of ROS contribute crucially to the regulation of heart muscle development and maintenance of homeostasis [20]. However, under pathological conditions, its overabundance or unregulated production causes oxidative stress and leads to oxidative degradation of proteins and lipids, cellular abnormality, DNA damage, mitochondrial dysfunctionality, and the subsequent irreversible cell death (apoptosis) [3]. Thus, in-depth insights into the formation of ROS-generated oxidative stress should be gained for the prognosis of HF in a better way and pharmacologically target the ROS-induced pathways for effective treatment outcomes in HF patients.

Various preclinical and clinical studies have indicated the involvement of increased ROS generation in the pathogenesis of HF via antioxidant deficit [21], increased oxidative stress [21-23], increased pericardial levels of 8-iso-PGF(2alpha) [24], and mitochondrial dysfunction [25]. Cytosolic sources of ROS [NADPH oxidase 4 (NOX4)] are implicated in increasing myocardial angiogenesis, an important facet of determining the adaptation of the heart to overload stress [26]. However, recent studies in HF models suggest alternative splicing of NOX4 mRNA, resulting in upregulating of full-length NOX4 in HF, potentially contributing to an increased ROS production [27]. Alternatively, other studies have shown that the reduction of NOX4 results in a decrease in the swelling of mitochondria and mitochondrial DNA damage [28]. Such contradictory findings have led to many uncertainties regarding the link between the role of ROS-generated oxidative stress as well as HF.

In light of this, conducting a systematic review that compiles high-quality evidence from peer-reviewed journals is crucial to explore the role of oxidative stress in inducing HF. Hence, the present study aims to systematically identify and analyze the role and implications of oxidative stress in HF, consolidating existing evidence linking oxidative stress markers and sources of oxidative stress production with the progression of HF. This endeavor also aims to map out further research possibilities in this domain. Besides, this systematic review aims to understand the potential role of biomarkers of oxidative stress as a target to prevent HF. The emphasis lies in terms of the context so that the clinical outcomes may be improved in patients diagnosed with HF, with a primary focus on the oxidative stress biomarker and its association with HF.

## Review

1. Methodology

A systematic review of the relevant existing studies was performed based on the Preferred Reporting Items for Systematic Reviews and Meta-Analyses (PRISMA) guidelines. The PRISMA procedures and checklist were followed in the present study [29].

1.1. Search Strategy

A systematic approach was undertaken to conduct the present study. The first step involved searching databases by using certain keywords. Major electronic databases, such as PubMed, Embase, Cochrane, and Sage were thoroughly searched, to find articles related to the association between the generation of free radicals and the progression and/or occurrence of HF. No restrictions were set on the publication date. The following MeSH terms were used in the search: "oxygen radicals, reactive oxygen species, free radical, reactive sulfur species, RSS, ROS, RNS, reactive nitrogen species, oxidative, antioxidants, redox, reactive, nitrative stress, species, oxidative stress, oxidant stress, oxidative damage, nitrative damage, antioxidative stress, antinitrative stress, antioxidant stress, heart failure, HF, CHF, heart failure, chronic heart failure with reduced ejection fraction, heart failure with preserved ejection fraction, HFpEF, HFrEF, association, relationship, predict, risk factor, determinant, progression, incidence, comparative study, cohort study, cross-sectional study, and case-control study". The Boolean words "OR" and "AND" were used. A detailed list of the search terms is presented below:

(‘free radical’ OR ‘oxygen radical’ OR ‘reactive oxygen species’ OR ‘ROS’ OR ‘reactive sulfur species’ OR ‘RSS’ OR ‘reactive nitrogen species’ OR ‘RNS’ OR ‘oxidative’ OR ‘antioxidants’ OR ‘redox’ OR ‘reactive’ OR ‘species’ OR ‘oxidative stress’ OR ‘oxidant stress’ OR ‘nitrative stress’ OR ‘oxidative damage’ OR ‘nitrative damage’ OR ‘antioxidative stress’ OR ‘antioxidant stress’ OR ‘antinitrative stress’) AND (‘heart failure’ OR ‘HF’ OR ‘chronic heart failure’ OR ‘CHF’ OR ‘heart failure with reduced ejection fraction’ OR ‘HFrEF OR ‘heart failure with preserved ejection fraction’ OR ‘HFpEF’) AND (‘association’ OR ‘relationship’ OR ‘predict’ OR ‘risk factor’ OR ‘determinant’) AND (‘comparative study’ OR ‘cross-sectional study’ OR ‘cohort study’ OR ‘case-control study’). The search initially yielded a total of 1,808 articles (Table 1). The details related to the search terms are provided in Appendix 1.

**Table 1:** Number of hits in various search databases

Databases	Number of hits
PubMed	545
Cochrane	804
Embase	429
Sage	30
Total	1,808

1.2. Eligibility (Inclusion and Exclusion) Criteria

A stepwise selection of the obtained 144 articles was performed based on the relevance of titles, abstracts, assessment of the main body, and close examination against the following preset inclusion criteria: (i) case-control and cohort studies (retrospective and prospective) with patients’ first diagnosis of HF and documented levels of oxidative stress or antioxidant markers or enzymes responsible for ROS production, (ii) studies that evaluated the role of oxidative stress in the progression and/or occurrence of HF, (iii) Articles that are available in the English language.

In order to minimize the possibility of any confounding effect that might affect the association between generation of free radicals and progression of HF, the following exclusion criteria were set: (i) case-control or cross-sectional studies conducted on children, adolescents, pregnant females, recipients of heart transplantation and animals were excluded to minimize the reverse causation; (ii) studies examining common risk factors such as obesity, hypertension, diabetes, dyslipidemia, etc., and not the role of free radical generation in the HF progression were excluded; (iii) randomized clinical trials assessing the action of medications on the free radical generation in HF patients were excluded; (iv) studies addressing cardiovascular diseases other than HF and studies focusing on mortality as an outcome were excluded; (v) book chapters, literature reviews, proceedings, records, systematic reviews, case reports, editorial,s and in vitro studies were excluded; and (vi) studies in which patients were already known to have definite HF were excluded.

1.3. Data Extraction

**Figure 1:**
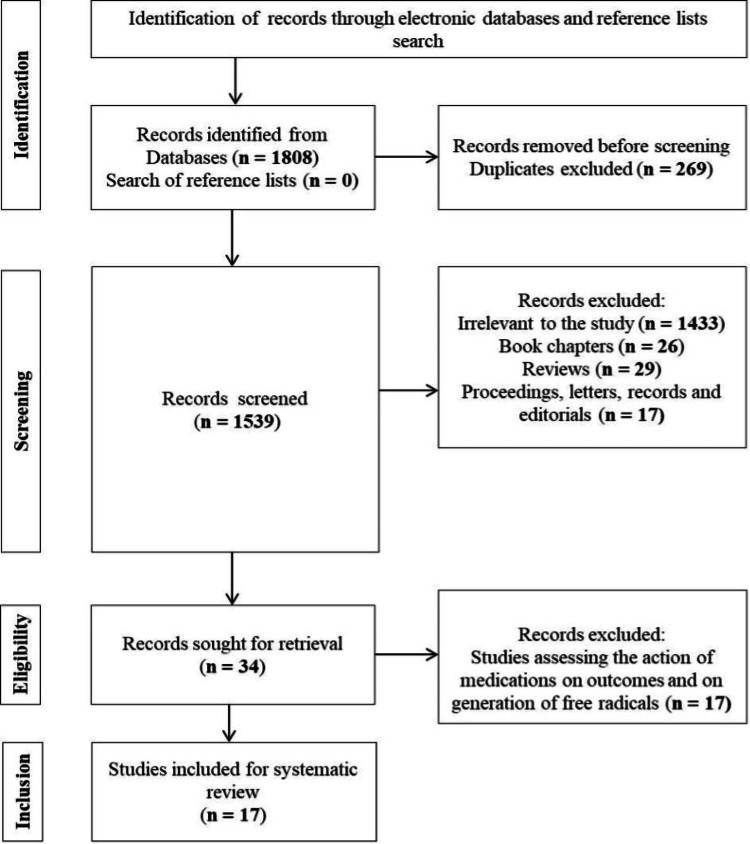
PRISMA chart depicting the step-wise selection of articles PRISMA: Preferred Reporting Items for Systematic Reviews and Meta-Analyses

To retrieve and summarize the relevant information from every single study, the data on the following variables were extracted: author(s), year of publication, type of study, population type and population size, subgroups (if any), mean age, male-to-female ratio, diagnostic method, oxidative stress markers or antioxidant markers or enzymes involved in ROS generation, major clinical outcomes (cardiovascular), and conclusive findings. Figure 1 shows the PRISMA chart depicting the selection process.

2. Results and discussion

2.1. Selection of Studies

Electronic search results on databases (PubMed, Embase, Cochrane, and Sage) and other sources initially yielded 1,808 articles for this study (Figure 1). Among the 1,539 screened articles, 34 articles were chosen for full-text assessment. A total of 17 studies were included in this review based on the implementation of the eligibility criteria. 

2.2. Descriptive Characteristics

A descriptive analysis of studies on the interrelation between oxidative stress and HF progression is crucial for reporting the trends, strengths, and limitations of the existing literature. This sub-section reviews the selected studies’ technical features (e.g., publication year, study design, and publication journals) and population features (e.g., sample size, age, gender, types of HF, and HF characterization), which provide an important basis for capturing the nature of this field of study and for recognizing the possible gaps that require a substantial consideration.

2.2.1. Distribution of studies (publication year): Given that the domain is receiving increasing attention [6], the oldest study included in this review was published in 2001. In the 14 years between 2001 and 2014, the pace of the generation of information relevant to the present research topic increased gradually: 52.9% of all studies included in this review. The number of studies that dealt with the association between oxidative stress and progression to HF increased further as the topic gathered more attention over the last six years, indicating that the included articles were pertinent and contemporary. In particular, three articles were published from 2017 to 2019, three articles in 2020, one article in 2021, and one article in 2022, collectively representing about 47% of the pool of selected studies. The recent development of research interest reflects the increasing practice of targeting oxidative stress in treating CHF and the growing attention towards this association. It is noteworthy that although the generation of academic information has peaked from 2019 onwards, data for 2022 is incomplete since we included publications before the completion of this study (October 2022). Therefore, it seems reasonable to assume that more publications will be available by the end of 2022.

**Figure 2:**
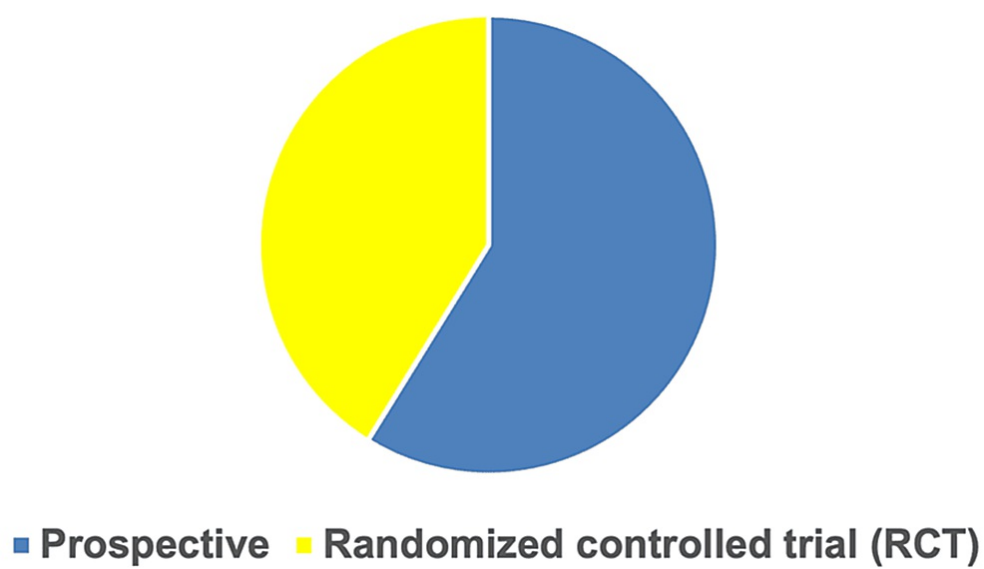
Proportion of study types in the review

2.2.2. Study design: Most of the 17 articles reviewed were prospective and case‑control studies and compared levels of oxidative stress or antioxidant markers in HF patients or enzymes involved in HF pathogenesis with age- and sex-matched controls. As illustrated in Figure 2, a majority involved prospective cohort studies (58.9%; n=10), whereas randomized controlled studies accounted for 41.1% (n=7). This finding reveals that the current state of research on the association between oxidative stress production and progression to HF needs more experimental contributions. Such contributions are crucial for a complete understanding of the research topic and for establishing a knowledge base. Thus, further experimental research is needed to improve the evidence base of this research domain.

2.2.3. Sample size: The population size in the studies ranged from 22 [30] and 774 [23,31]. We incorporated 4,487 patients: 4,168 with HF and 319 healthy control subjects. The number of patients with HF varied between 22 [30] and 774 [23,31].

2.2.4. Age: The mean age group among the HF patients was 61.1 ± 6.7 years (range: 45.5 ± 6.5 [32] to 72.6 ±11 [33]) while it was 56.0 ± 6.7 years in the control group (range: 47.0 ± 4.4 [30] to 69.2 ± 10 [34]). Eight studies followed a prospective approach and did not include any control groups.

2.2.5. Gender: The total numbers of males and females in the included studies were 3018 and 1329, respectively. The number of males varied between 12 [35,36] and 664 [31] in the HF group and from five [35,36] and 249 [23] in the control group. One study did not specify the male-to-female ratio [30].

**Figure 3:**
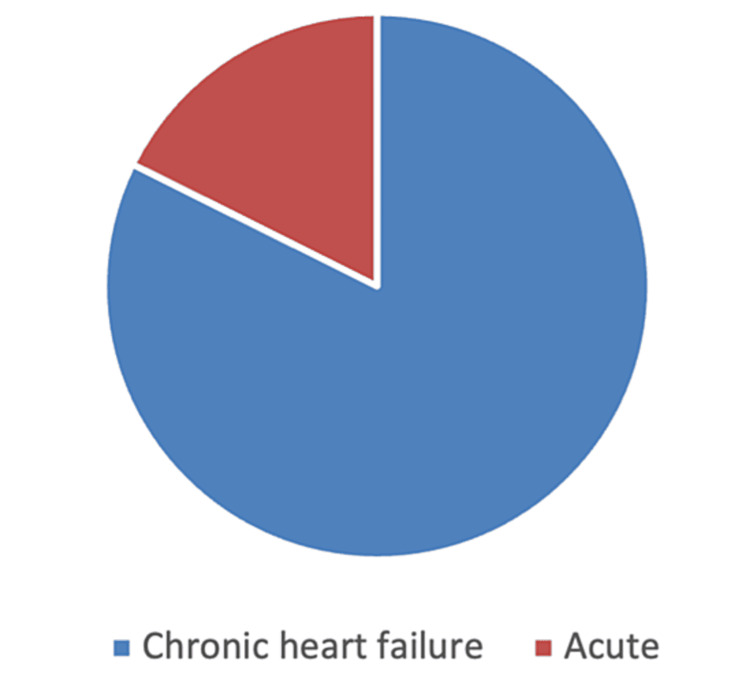
Types of HF examined HF: heart failure

2.2.6. Types of HF studied: The findings of this study (Figure 3) indicate that CHF was the most studied type of HF (82.3%; n=14). This was not surprising, given the conditions and associated adverse outcomes of CHF. Patients hospitalized with acute myocardial infarction (MI) made up the second most examined population (17.6%, n=3). Chronic HFrEF accounted for 28.5% of the studies (n=4), followed by CHF with dilated cardiomyopathy (DCM) (n=3; 21.4%), ischemic cardiomyopathy (ICM; n=2; 14.2%), and HFpEF (n=1; 7.1%). We believe that further studies are needed to examine other types of HF because clinical management practices might be influenced by the nature of the disease.

2.2.7. Characterization of markers: Studies evaluated different biomarkers to assess the oxidative stress in the patient/treated group versus control groups. Among the total of 17 studies, 14 reported the association between HF and different types of oxidative stress marker levels [oxidized (ox) low-density lipoprotein (LDL)/biopyrrins/8-epi-PGF(2alpha)/malondialdehyde (MDA)/reactive carbonyl derivatives (RCD)/protein thiol groups (PS-H)/uric acid (UA) concentration/derivative of reactive oxygen metabolite (DROM)/interleukins 10 (IL10)/highly-sensitive C-reactive protein (hs-CRP)/oxidative stress index (OSI)/total oxidant status (TOS)/glutathione peroxidase (GSH-Px)/xanthine oxidoreductase (XOR)/superoxide dismutase (SOD)/glutathione peroxidase (GPX)/myeloperoxidase (MPO)/paraoxonase 3 (PON3) (1362 cases and 1168 controls)] (Table 2) [23,30-42]. Eight of the 14 studies reported an association among the different levels and types of enzyme sources of ROS and HF [30,33,36-40,42]. Furthermore, six studies reported the association between HF and antioxidant marker levels [edavarone/oxypurinol/coenzyme Q_10_ (CoQ_10_)/total antioxidant capacity (TAC)] (Table 2) [23,31,38,43-45].

**Table 2:** Summary of the included studies 1: author (year of publication); 2: study type; 3: population type/size; 4: subgroups; 5: mean age; 6: male/female; 7: diagnostic method; 8: oxidative stress markers/ enzymes involved in ROS generation treatment protocol; 9: mean observation period; 10: clinical (major cardiovascular) outcomes; 11: conclusive findings RCT: randomized controlled trial; HFrEF: heart failure with reduced ejection fraction; DCM: dilated cardiomyopathy; ICM: ischemic cardiomyopathy; HFpEF: heart failure with preserved ejection fraction; CHF: chronic heart failure; STEMI: ST-elevation myocardial infarction; NSTEMI: non-ST-elevation myocardial infarction; AMI: acute myocardial infarction; XOR: xanthine oxidoreductase; TIMI: thrombolysis in myocardial infarction; EF: ejection fraction; CoQ_10_: coenzyme Q_10_; nICM: non-ischaemic cardiomyopathy; ECG: electrocardiogram; NYHA: The New York Heart Association; OSI: oxidative stress index; TOS: total oxidant status; MDA: malondialdehyde; TAC: total antioxidant capacity; NADPH: nicotinamide adenine dinucleotide phosphate; UA: uric acid; DROM: derivative of reactive oxygen metabolite; oxLDL: oxidized low-density lipoprotein; SOD: superoxide dismutase; GPX: glutathione peroxidase; MPO: myeloperoxidase; IL10: interleukin 10; hs-CRP: highly-sensitive C-reactive protein; PON3: paraoxonase 3; RCD: reactive carbonyl derivatives; GSH-Px: glutathione peroxidase; PS-H: protein thiol groups; CK-MB: creatine kinase-MB isoenzymes

1	2	3	4	5	6	7	8	9	10	11
Romuk et al. (2019) [23]	Prospective	Patients with HFrEF (n=774)	Patients with ICM (n=479), non-ICM patients (n=295)	ICM (56.4 ± 8.0), nICM (47.4 ± 11.7)	ICM (416/63), nICM (249/46)	Physical checkup, ECG, and echocardiography	OSI, TOS, MDA, and TAC	180 days	ICM patients had lower levels of serum OSI and higher MDA and TAC concentrations	A relationship between oxidative stress and HF severity was confirmed
Heymes et al. (2003) [30]	Prospective	Patients with end-stage HF (n=13), explanted nonfailing heart (n=9)	NR	Patients with end-stage HF (53 ± 3.2), controls (47 ± 4.4)	NR	NR	NADPH oxidase	7 days	NADPH oxidase activity was significantly higher in failing hearts vs. non‑failing hearts	The increase in NADPH oxidase in the end-stage of HF may indicate the involvement of oxidative stress in the progression to HF
Romuk et al. (2019) [31]	Prospective	Patients with chronic HFrEF (n=774)		54	664/110	Echocardiography	MDA, UA concentration, TOS, OSI, and TAC	365 days	Higher levels of serum TAC, MDA, and UA were associated with death risk due to HF	Among all OS markers, MDA and UA concentration were identified as significant predictors of heart transplantation and death due to worsening HF
Wolfram et al. (2005) [32]	RCT	Patients with DCM (n=20), patients with ICM (n=20), healthy age- and sex-matched controls (n=20)	NA	DCM (46 ± 7), ICM (45 ± 6), controls (47 ± 6)	DCM (16/4), ICM (16/4), controls (16/4)	NYHA assessment	8-epi-PGF(2alpha)	14-60 days	Plasma, serum, and urine levels of 8-Epi-PGF(2alpha) were significantly higher in CM patients vs. controls, irrespective of confounders, and this increased with the progression of HF	The levels of 8-epi-PGF(2alpha) were positively associated with the disease severity, implying the relevance of oxidative stress in the progression of HF
Watanabe et al. (2020) [33]	Prospective	Patients with HFpEF (n=257)	HFpEF patients with low XOR (n=45), HFpEF patients with normal XOR (n=160), HFpEF with high XOR (n=52)	72.6 ± 11	142/115	ECG	XOR	809 days	HFpEF with high XOR exhibited a higher risk for adverse cardiovascular outcomes like re‑hospitalization and cardiac death due to progressive HF, irrespective of the presence of comorbidities including hyperuricemia	High levels of plasma XOR activity were significantly associated with adverse cardiovascular outcomes in individuals with HFpEF
Nishihara et al. (2020) [34]	RCT	Hospitalized patients with HFrEF (n=114), age-, sex-and risk factors-matched non-HF patients (n=114)	NA	HFrEF group (69.2 ± 9.7), non-HF group (69.2 ± 10)	HFrEF group (72/42), non-HF group (70/44)	ECG	DROM	638 days	Serum DROM was significantly higher in the HFrEF group than in the control group. Hospitalization for decompensated HF was recorded and this was higher in the group with high DROM levels than the low-DROM group	A significant association between DROM and HF-associated events was identified
Tsutamoto et al. (2001) [35]	Prospective	Patients with mild CHF and DCM (n=22), age-matched controls (n=8)	NA	CHF patients (57 ± 4.0), controls (54 ± 4.5)	CHF patients (12/10), controls (5/3)	Clinical records, physical checkups, ECG, echocardiography, chest radiography, left ventriculography, and coronary angiography	oxLDL	60 days	The plasma level of oxLDL was significantly higher in patients with DCM than in controls	oxLDL-induced oxidative stress may lead to left ventricular abnormality in DCM patients
Maack et al. (2003) [36]	Prospective	Patients with DCM (n=8), patients with ICM (n=8), controls (n=8)	NA	DCM (54 ± 4), ICM (55 ± 3), controls (49 ± 5)	DCM (5/3), ICM (7/1), controls (5/3)	ECG	NADPH oxidase, aconitase, and lipid hydroperoxides	28 days	NADPH oxidase activity was significantly higher in DCM or ICM patients (1.5-fold) compared with controls. Failing myocardium of DCM and ICM patients had significantly reduced aconitase activity and lipid hydroperoxides (higher levels) than controls	Oxidative stress-induced increase of Ras-related C3 botulinum toxin substrate 1 (rac1)-GTPase activity was observed in DCM and ICM patients
Otaki et al. (2017) [37]	Prospective	Patients admitted for diagnosis or treatment of CHF (n=440), age- and sex-matched controls (n=44)	CHF patient with low XOR (n=57), normal XOR (n=268), high XOR (n=115)	Patients (70 ±12), controls (67 ±12)	Patients (269/171), controls (23/21)	ECG	XOR	1,034 days	158 cardiac events (re‑hospitalization and cardiac death due to progressive HF) were observed after adjusting for confounders. This rate was higher in the high XOR group followed by the low XOR group	Both high and low plasma XOR activities were significantly associated with the severity of CHF, indicating it as an independent risk factor for CHF
Matin et al. (2020) [38]	Prospective	STEMI patients (n=200)	Patients with low TIMI flow (n=100), patients with high TIMI flow (n=100)	60.7 ± 11.8	153/47	ECG and chest X-ray	SOD, MDA, GPX, and TAC	6 hours	Serum SOD and GPX levels were significantly higher in group B than in group A. In addition, HF post-MI was higher in group A compared to the other group	SOD and GPX were significant predictors of post-MI HF in STEMI patients
Roumeliotis et al. (2021) [39]	Prospective	NSTEMI patients (n=100)	NSTEMI patients with high EF (n=57), patients with low EF (n=43)	66.5 ± 11.4	49/51	Clinical records, ECG echocardiography, coronary angiography, and biochemical assessments	MPO, IL10, and hs-CRP	1-3 days	Patients with low EF had higher concentrations of serum MPO, IL10, and hs-CRP, which further decreased with the progression of HF	The oxidative stress markers were identified as significant predictors of severe cardiac events, including HF
Klimczak-Tomaniak et al. (2022) [40]	Prospective	Patients with HFrEF (n=250)		67	184/66	Echocardiography	PON3	803 days	Patients (n=53) who were re‑hospitalized for worsening HF had high levels of plasma PON3 compared to patients who were free from the primary outcome	PON3 was confirmed to be associated with the occurrence of adverse clinical events in HFrEF patients
Hokamaki et al. (2004) [41]	RCT	HF patients (n=94), age-matched controls (n=47)	NA	HF patents (65 ± 1), controls (65 ± 2)	HF patients (59/35), controls (30/17)	Clinical records, ECG, echocardiogram, chest X-ray, left ventriculogram, and coronary angiogram	Biopyrrins	A few hours to 1 day	The urinary biopyrrin levels were significantly higher in the HF patients (maximum in class III/IV NYHA compared to the controls	Urinary biopyrrin levels were higher in HF patients and this level increased along with its severity
Radovanovic et al. (2012) [42]	RCT	Patients with CHF (n=169), age- and sex-matched healthy controls (n=69)		CHF patients (59.0 ± 5.3), controls (58.4 ± 5.5)	CHF patients (74/46)	Clinical records, physical checkups, ECG, echocardiography, chest radiology, and coronary angiography	Plasma MDA, RCD, SOD, GSH-Px, and PS-H	13.1 months	A weak but significant correlation was found between GPX and the occurrence of cardiovascular events, including CHF-related hospitalization. MDA was identified as an independent predictor of hospitalization for worsening HF and death due to HF	Carbonyl stress is involved in the course of progression to CHF
Tsujita et al. (2004) [43]	RCT	Patients with initial AMI (n=80)	Edaravone (n=40), saline (n=40)	Edaravone (63 ± 2), saline (62 ± 2)	31/49	ECG	10 minute-IV administration of 30 mg edaravone prior to reperfusion and saline as placebo	6, 12, 18, and 24 hours, and on 3, 7, and 14 days (before and after reperfusion)	The area under the serum CK-MB curve and the peak CK-MB was significantly lower in the edaravone group than in the control group	An association and better clinical outcomes were observed between the administration of edaravone before myocardial reperfusion and smaller infarcts
Hare et al. (2008) [44]	RCT	Patients with symptomatic HF (n=405)	Patients receiving oxypurinol (n=203), placebo (n=202)	Oxypurinol (64 ± 13), placebo (65 ± 13)	Oxypurinol (76/127), placebo (70/132)	Clinical records, physical checkups, NYHA functional class assessment	6 capsules of oxypurinol (600 mg/day) for both groups during the first week after randomization	168 days	A higher hospitalization rate due to worsening of HF was found in the oxypurinol group compared to the placebo group; however, the difference was statistically insignificant	No oxypurinol-induced clinical improvement was observed in unselected moderate-to-severe HF patients
Mortensen et al. (2014) [45]	RCT	Patients with moderate-severe CHF (n=420)	CoQ_10_ (n=202), placebo (n=218)	CoQ_10_ (62.3 ± 12), placebo (62.3 ± 11)	CoQ_10_ (154/48), placebo (151/67)	ECG, NYHA assessment	CoQ_10_	730 days	The CoQ_10 _group had a significantly higher level of serum CoQ_10 _than the placebo group at 112 days. Incidence for hospitalization due to HF was significantly lower in the CoQ_10 _group vs. the placebo group	Long-term treatment of HF patients with CoQ_10_ reduces adverse cardiovascular outcomes

2.3. Content Analysis

2.3.1. Oxidative stress marker and HF: The markers of oxidative stress and their clinical level were evaluated in plasma in six studies [32,33,35,37,40,42], in serum in six studies [23,31,32,34,38,39] and urine in two studies [32,41]. In some of the reviewed studies, total oxidative stress levels were assessed by measuring the endogenous levels of organic GSH-Px, XOR, SOD, GPX, MPO, and PON3 that were related to the production of free radicals [30,33,36-40,42].

Many researchers have indicated the involvement of MDA in the course of HF progression [23,31,38,42]. Most notably, Radovanovic et al. [42] identified MDA as an independent predictor of adverse cardiovascular outcomes due to HF, such as hospitalization (due to worsening of the condition) and eventual death. Romuk et al. [23] reported that higher serum MDA levels were found to be related to the severity of HF in ICM patients. Further, Romuk et al. [23] confirmed that elevated levels of serum MDA were related to mortality fear caused by HF. Most of the relevant studies examining the association among oxidative stress generation, clinical outcomes, and severity of HF revealed an elevated level of lipid peroxidation and MDA in the plasma of patients with moderate-to-severe HF with ICM and DCM [46,47]. Similarly, scholars have shown that XOR [33,37] is one of the main enzymes accountable for the excessive production of ROS in CHF patients or patients with HFpEF. The study by Otaki et al. [37] showed that higher levels of XOR were found to be associated with higher incidences of cardiac events, such as re‑hospitalization and death caused by progressive and chronic HF.

The study by Watanabe et al. [33] investigated XOR levels in patients with HFpEF and found a significant correlation between progressive HF-induced re‑hospitalization and cardiac death caused by MDA and high XOR levels in HFpEF patients. Moreover, Matin et al. [38] stated that the extent of serum SOD and GPX were significantly higher in STEMI patients with high TIMI flow after percutaneous coronary intervention (PCI). Klimczak-Tomaniak et al. [40] indicated the association between elevated levels of plasma PON3 and re‑hospitalized patients with worsening HF. Furthermore, other markers were reported in only one study. The study by Tsutamoto et al. [35] showed that the levels of oxLDL in plasma were interrelated with the occurrence of HF. Hokamaki et al. [41] investigated urinary biopyrrins levels in severe HF patients and found a significant correlation between the two. A clinical study in cardiomyopathy patients showed a high amount of 8-epi-PGF(2alpha) in plasma, serum, and urine when compared to healthy controls, which further increased as the condition progressed (32). However, Romuk et al. [23] indicated the association between HF-induced death risk and elevated levels of serum TAC and UA concentration in HFrEF patients. Nishihara et al. [34] showed that HFrEF patients had higher levels of serum DROM vs. controls. Similarly, in non-ST-elevation MI patients with low EF, serum concentrations of MPO, IL 10, and hs-CRP were increased [39].

2.3.2. Antioxidant markers and HF: Levels of antioxidant markers were evaluated in six studies [23,31,38,43-45]. The second noticeable research domain stemming from the reviewed studies was the antioxidant marker levels and the associated clinical outcomes in HF patients. As reported by Tsujita et al. [43], the area under the serum creatine kinase-MB isoenzymes (CK-MB) curve and the peak CK-MB was found to be significantly lower among the patients having acute MI who were treated with edaravone when compared to the control group. In the same vein, Mortensen et al. [45] demonstrated that when moderate-to-severe chronic HF patients were treated with the CoQ_10_ group, their incidence of hospitalization caused by HF was found to be significantly lower than the placebo group. Studies [23,31] have also demonstrated that TAC concentrations were higher in ICM patients, which, in turn, were predictive of HF-induced mortality. However, a different opinion was expressed in the study by Hare et al. [44] where treatment with oxypurinol resulted in no significant difference in re‑hospitalization incidences due to the worsening of HF. Similarly, Matin et al. [38] demonstrated no significant contrast between STEMI patients having low and high TIMI flow in terms of their TAC levels. Thus, the connection between the extent of antioxidant markers in the body and the progression to HF requires further clinical assessments.

Limitations

This review has a few limitations. The selected studies were performed at a single center and a few of the studies had a small patient population. Thus, to validate the prognostic value of oxidative stress maker as an independent predictor of HF, more investigations with larger sample sizes and involving multicenters are required. Furthermore, future studies should involve longer follow-ups to evaluate the changes in the oxidative stress biomarkers over time.

## Conclusions

The significant growth in the literature regarding the functional role of oxidative stress generation in causing clinical outcomes and severity in HF over the past few years motivated us to explore whether considering oxidative stress markers or antioxidant markers can be an effective targeting strategy for devising better management practices for CHF. Our findings show that the oxidative stress level or antioxidant markers or enzymes involved in ROS generation are associated with outcomes in the progression of HF. Given the increased occurrence of CHF and the current awareness that its association with oxidative stress production tends to strengthen, we believe these findings are robust.

Our findings have significant implications for clinicians. There are established predictors such as LVEF and NYHA classification that are widely used to assess HF; however, these are not adequate to risk-stratify HF patients. Therefore, based on the findings of this systematic review, multiple oxidative stress markers could be used as a potential prognostic tool to assess HF. Furthermore, the identification of oxidative stress markers can be useful for therapy-related decision-making. Clinicians can create a panel of oxidative stress markers and assign a score to each oxidative stress marker to improve risk prediction in HF patients. In this sense, a single oxidative marker or a combination of oxidative markers can be used to assess the outcomes in HF patients, including the duration of HF and risk stratification of HF patients. For instance, the presence of elevated levels of MDA and UA levels can be used for risk stratification of chronic HF patients. The non-invasive nature, reproducibility, quantitative measuring, and rapid availability are the major advantages of oxidative stress marker laboratory tests. Thus, from a clinical perspective, health centers should implement the assessment of oxidative stress markers as a routine clinical test to assess HF prognosis, which is critical for the clinical management of HF.

## Appendices

**Table 3:** Search databases, sample search queries, and number of hits

Search database	Sample search queries		No. of hits
PubMed	1	(‘Free radical’ OR ‘oxygen radical’ OR ‘reactive oxygen species’ OR ‘ROS’ OR ‘reactive sulfur species’ OR ‘RSS’ OR ‘reactive nitrogen species’ OR ‘RNS’ OR ‘oxidative’ OR ‘antioxidants’ OR ‘redox’ OR ‘reactive’ OR ‘species’ OR ‘oxidative stress’ OR ‘oxidant stress’ OR ‘nitrative stress’ OR ‘oxidative damage’ OR ‘nitrative damage’ OR ‘antioxidative stress’ OR ‘antioxidant stress’ OR ‘antinitrative stress’)	51,980
	2	(‘Free radical’ OR ‘oxygen radical’ OR ‘reactive oxygen species’ OR ‘ROS’ OR ‘reactive sulfur species’ OR ‘RSS’ OR ‘reactive nitrogen species’ OR ‘RNS’ OR ‘oxidative’ OR ‘antioxidants’ OR ‘redox’ OR ‘reactive’ OR ‘species’ OR ‘oxidative stress’ OR ‘oxidant stress’ OR ‘nitrative stress’ OR ‘oxidative damage’ OR ‘nitrative damage’ OR ‘antioxidative stress’ OR ‘antioxidant stress’ OR ‘antinitrative stress’) AND (‘Heart failure’ OR ‘HF’ OR ‘chronic heart failure’ OR ‘CHF’ OR ‘heart failure with reduced ejection fraction’ OR ‘HFrEF OR ‘heart failure with preserved ejection fraction’ OR ‘HFpEF’)	1,401
	3	(‘Free radical’ OR ‘oxygen radical’ OR ‘reactive oxygen species’ OR ‘ROS’ OR ‘reactive sulfur species’ OR ‘RSS’ OR ‘reactive nitrogen species’ OR ‘RNS’ OR ‘oxidative’ OR ‘antioxidants’ OR ‘redox’ OR ‘reactive’ OR ‘species’ OR ‘oxidative stress’ OR ‘oxidant stress’ OR ‘nitrative stress’ OR ‘oxidative damage’ OR ‘nitrative damage’ OR ‘antioxidative stress’ OR ‘antioxidant stress’ OR ‘antinitrative stress’) AND (‘Heart failure’ OR ‘HF’ OR ‘chronic heart failure’ OR ‘CHF’ OR ‘heart failure with reduced ejection fraction’ OR ‘HFrEF OR ‘heart failure with preserved ejection fraction’ OR ‘HFpEF’) AND (‘association’ OR ‘relationship’ OR ‘predict’ OR ‘risk factor’ OR ‘determinant’)	1,197
	4	(‘Free radical’ OR ‘oxygen radical’ OR ‘reactive oxygen species’ OR ‘ROS’ OR ‘reactive sulfur species’ OR ‘RSS’ OR ‘reactive nitrogen species’ OR ‘RNS’ OR ‘oxidative’ OR ‘antioxidants’ OR ‘redox’ OR ‘reactive’ OR ‘species’ OR ‘oxidative stress’ OR ‘oxidant stress’ OR ‘nitrative stress’ OR ‘oxidative damage’ OR ‘nitrative damage’ OR ‘antioxidative stress’ OR ‘antioxidant stress’ OR ‘antinitrative stress’) AND (‘Heart failure’ OR ‘HF’ OR ‘chronic heart failure’ OR ‘CHF’ OR ‘heart failure with reduced ejection fraction’ OR ‘HFrEF OR ‘heart failure with preserved ejection fraction’ OR ‘HFpEF’) AND (‘association’ OR ‘relationship’ OR ‘predict’ OR ‘risk factor’ OR ‘determinant’) AND (‘comparative study’ OR ‘cross-sectional study’ OR ‘cohort study’ OR ‘case-control study’).	545
Cochrane	1	(‘Free radical’ OR ‘oxygen radical’ OR ‘reactive oxygen species’ OR ‘ROS’ OR ‘reactive sulfur species’ OR ‘RSS’ OR ‘reactive nitrogen species’ OR ‘RNS’ OR ‘oxidative’ OR ‘antioxidants’ OR ‘redox’ OR ‘reactive’ OR ‘species’ OR ‘oxidative stress’ OR ‘oxidant stress’ OR ‘nitrative stress’ OR ‘oxidative damage’ OR ‘nitrative damage’ OR ‘antioxidative stress’ OR ‘antioxidant stress’ OR ‘antinitrative stress’)	86,792
	2	(‘Free radical’ OR ‘oxygen radical’ OR ‘reactive oxygen species’ OR ‘ROS’ OR ‘reactive sulfur species’ OR ‘RSS’ OR ‘reactive nitrogen species’ OR ‘RNS’ OR ‘oxidative’ OR ‘antioxidants’ OR ‘redox’ OR ‘reactive’ OR ‘species’ OR ‘oxidative stress’ OR ‘oxidant stress’ OR ‘nitrative stress’ OR ‘oxidative damage’ OR ‘nitrative damage’ OR ‘antioxidative stress’ OR ‘antioxidant stress’ OR ‘antinitrative stress’) AND (‘Heart failure’ OR ‘HF’ OR ‘chronic heart failure’ OR ‘CHF’ OR ‘heart failure with reduced ejection fraction’ OR ‘HFrEF OR ‘heart failure with preserved ejection fraction’ OR ‘HFpEF’)	2,537
	3	(‘Free radical’ OR ‘oxygen radical’ OR ‘reactive oxygen species’ OR ‘ROS’ OR ‘reactive sulfur species’ OR ‘RSS’ OR ‘reactive nitrogen species’ OR ‘RNS’ OR ‘oxidative’ OR ‘antioxidants’ OR ‘redox’ OR ‘reactive’ OR ‘species’ OR ‘oxidative stress’ OR ‘oxidant stress’ OR ‘nitrative stress’ OR ‘oxidative damage’ OR ‘nitrative damage’ OR ‘antioxidative stress’ OR ‘antioxidant stress’ OR ‘antinitrative stress’) AND (‘Heart failure’ OR ‘HF’ OR ‘chronic heart failure’ OR ‘CHF’ OR ‘heart failure with reduced ejection fraction’ OR ‘HFrEF OR ‘heart failure with preserved ejection fraction’ OR ‘HFpEF’) AND (‘association’ OR ‘relationship’ OR ‘predict’ OR ‘risk factor’ OR ‘determinant’)	1,619
	4	(‘Free radical’ OR ‘oxygen radical’ OR ‘reactive oxygen species’ OR ‘ROS’ OR ‘reactive sulfur species’ OR ‘RSS’ OR ‘reactive nitrogen species’ OR ‘RNS’ OR ‘oxidative’ OR ‘antioxidants’ OR ‘redox’ OR ‘reactive’ OR ‘species’ OR ‘oxidative stress’ OR ‘oxidant stress’ OR ‘nitrative stress’ OR ‘oxidative damage’ OR ‘nitrative damage’ OR ‘antioxidative stress’ OR ‘antioxidant stress’ OR ‘antinitrative stress’) AND (‘Heart failure’ OR ‘HF’ OR ‘chronic heart failure’ OR ‘CHF’ OR ‘heart failure with reduced ejection fraction’ OR ‘HFrEF OR ‘heart failure with preserved ejection fraction’ OR ‘HFpEF’) AND (‘association’ OR ‘relationship’ OR ‘predict’ OR ‘risk factor’ OR ‘determinant’) AND (‘comparative study’ OR ‘cross-sectional study’ OR ‘cohort study’ OR ‘case-control study’)	804
Embase	1	(‘Free radical’ OR ‘oxygen radical’ OR ‘reactive oxygen species’ OR ‘ROS’ OR ‘reactive sulfur species’ OR ‘RSS’ OR ‘reactive nitrogen species’ OR ‘RNS’ OR ‘oxidative’ OR ‘antioxidants’ OR ‘redox’ OR ‘reactive’ OR ‘species’ OR ‘oxidative stress’ OR ‘oxidant stress’ OR ‘nitrative stress’ OR ‘oxidative damage’ OR ‘nitrative damage’ OR ‘antioxidative stress’ OR ‘antioxidant stress’ OR ‘antinitrative stress’)	36,754
	2	(‘Free radical’ OR ‘oxygen radical’ OR ‘reactive oxygen species’ OR ‘ROS’ OR ‘reactive sulfur species’ OR ‘RSS’ OR ‘reactive nitrogen species’ OR ‘RNS’ OR ‘oxidative’ OR ‘antioxidants’ OR ‘redox’ OR ‘reactive’ OR ‘species’ OR ‘oxidative stress’ OR ‘oxidant stress’ OR ‘nitrative stress’ OR ‘oxidative damage’ OR ‘nitrative damage’ OR ‘antioxidative stress’ OR ‘antioxidant stress’ OR ‘antinitrative stress’) AND (‘Heart failure’ OR ‘HF’ OR ‘chronic heart failure’ OR ‘CHF’ OR ‘heart failure with reduced ejection fraction’ OR ‘HFrEF OR ‘heart failure with preserved ejection fraction’ OR ‘HFpEF’)	1,230
	3	(‘Free radical’ OR ‘oxygen radical’ OR ‘reactive oxygen species’ OR ‘ROS’ OR ‘reactive sulfur species’ OR ‘RSS’ OR ‘reactive nitrogen species’ OR ‘RNS’ OR ‘oxidative’ OR ‘antioxidants’ OR ‘redox’ OR ‘reactive’ OR ‘species’ OR ‘oxidative stress’ OR ‘oxidant stress’ OR ‘nitrative stress’ OR ‘oxidative damage’ OR ‘nitrative damage’ OR ‘antioxidative stress’ OR ‘antioxidant stress’ OR ‘antinitrative stress’) AND (‘Heart failure’ OR ‘HF’ OR ‘chronic heart failure’ OR ‘CHF’ OR ‘heart failure with reduced ejection fraction’ OR ‘HFrEF OR ‘heart failure with preserved ejection fraction’ OR ‘HFpEF’) AND (‘association’ OR ‘relationship’ OR ‘predict’ OR ‘risk factor’ OR ‘determinant’)	810
	4	(‘Free radical’ OR ‘oxygen radical’ OR ‘reactive oxygen species’ OR ‘ROS’ OR ‘reactive sulfur species’ OR ‘RSS’ OR ‘reactive nitrogen species’ OR ‘RNS’ OR ‘oxidative’ OR ‘antioxidants’ OR ‘redox’ OR ‘reactive’ OR ‘species’ OR ‘oxidative stress’ OR ‘oxidant stress’ OR ‘nitrative stress’ OR ‘oxidative damage’ OR ‘nitrative damage’ OR ‘antioxidative stress’ OR ‘antioxidant stress’ OR ‘antinitrative stress’) AND (‘Heart failure’ OR ‘HF’ OR ‘chronic heart failure’ OR ‘CHF’ OR ‘heart failure with reduced ejection fraction’ OR ‘HFrEF OR ‘heart failure with preserved ejection fraction’ OR ‘HFpEF’) AND (‘association’ OR ‘relationship’ OR ‘predict’ OR ‘risk factor’ OR ‘determinant’) AND (‘comparative study’ OR ‘cross-sectional study’ OR ‘cohort study’ OR ‘case-control study’)	429
Sage	1	(‘Free radical’ OR ‘oxygen radical’ OR ‘reactive oxygen species’ OR ‘ROS’ OR ‘reactive sulfur species’ OR ‘RSS’ OR ‘reactive nitrogen species’ OR ‘RNS’ OR ‘oxidative’ OR ‘antioxidants’ OR ‘redox’ OR ‘reactive’ OR ‘species’ OR ‘oxidative stress’ OR ‘oxidant stress’ OR ‘nitrative stress’ OR ‘oxidative damage’ OR ‘nitrative damage’ OR ‘antioxidative stress’ OR ‘antioxidant stress’ OR ‘antinitrative stress’)	356
	2	(‘Free radical’ OR ‘oxygen radical’ OR ‘reactive oxygen species’ OR ‘ROS’ OR ‘reactive sulfur species’ OR ‘RSS’ OR ‘reactive nitrogen species’ OR ‘RNS’ OR ‘oxidative’ OR ‘antioxidants’ OR ‘redox’ OR ‘reactive’ OR ‘species’ OR ‘oxidative stress’ OR ‘oxidant stress’ OR ‘nitrative stress’ OR ‘oxidative damage’ OR ‘nitrative damage’ OR ‘antioxidative stress’ OR ‘antioxidant stress’ OR ‘antinitrative stress’) AND (‘Heart failure’ OR ‘HF’ OR ‘chronic heart failure’ OR ‘CHF’ OR ‘heart failure with reduced ejection fraction’ OR ‘HFrEF OR ‘heart failure with preserved ejection fraction’ OR ‘HFpEF’)	55
	3	(‘Free radical’ OR ‘oxygen radical’ OR ‘reactive oxygen species’ OR ‘ROS’ OR ‘reactive sulfur species’ OR ‘RSS’ OR ‘reactive nitrogen species’ OR ‘RNS’ OR ‘oxidative’ OR ‘antioxidants’ OR ‘redox’ OR ‘reactive’ OR ‘species’ OR ‘oxidative stress’ OR ‘oxidant stress’ OR ‘nitrative stress’ OR ‘oxidative damage’ OR ‘nitrative damage’ OR ‘antioxidative stress’ OR ‘antioxidant stress’ OR ‘antinitrative stress’) AND (‘Heart failure’ OR ‘HF’ OR ‘chronic heart failure’ OR ‘CHF’ OR ‘heart failure with reduced ejection fraction’ OR ‘HFrEF OR ‘heart failure with preserved ejection fraction’ OR ‘HFpEF’) AND (‘association’ OR ‘relationship’ OR ‘predict’ OR ‘risk factor’ OR ‘determinant’)	30
	4	(‘Free radical’ OR ‘oxygen radical’ OR ‘reactive oxygen species’ OR ‘ROS’ OR ‘reactive sulfur species’ OR ‘RSS’ OR ‘reactive nitrogen species’ OR ‘RNS’ OR ‘oxidative’ OR ‘antioxidants’ OR ‘redox’ OR ‘reactive’ OR ‘species’ OR ‘oxidative stress’ OR ‘oxidant stress’ OR ‘nitrative stress’ OR ‘oxidative damage’ OR ‘nitrative damage’ OR ‘antioxidative stress’ OR ‘antioxidant stress’ OR ‘antinitrative stress’) AND (‘Heart failure’ OR ‘HF’ OR ‘chronic heart failure’ OR ‘CHF’ OR ‘heart failure with reduced ejection fraction’ OR ‘HFrEF OR ‘heart failure with preserved ejection fraction’ OR ‘HFpEF’) AND (‘association’ OR ‘relationship’ OR ‘predict’ OR ‘risk factor’ OR ‘determinant’) AND (‘comparative study’ OR ‘cross-sectional study’ OR ‘cohort study’ OR ‘case-control study’)	30
